# Genetic Variants and Anterior Cruciate Ligament Rupture: A Systematic Review

**DOI:** 10.1007/s40279-017-0678-2

**Published:** 2017-01-19

**Authors:** Mustafa Kaynak, Frank Nijman, Joyce van Meurs, Max Reijman, Duncan E. Meuffels

**Affiliations:** 1 Department of Orthopaedic Surgery, Erasmus MC, University Medical Center Rotterdam, ‘s-Gravendijkwal 230, 3000 CA Rotterdam, The Netherlands; 2 Department of Internal Medicine, Erasmus MC, University Medical Center Rotterdam, Rotterdam, The Netherlands

## Abstract

**Background:**

Studies have shown a familial predisposition for anterior cruciate ligament (ACL) rupture and have been followed by genetic-association studies on polymorphisms in candidate genes in recent years. To date, no systematic review with a best-evidence synthesis has evaluated the influence of genetics on this devastating knee injury.

**Objective:**

Our objective was to evaluate the association between genetic variants and ACL rupture.

**Methods:**

We performed an extensive search in Embase, MEDLINE, Web of Science, Scopus, PubMed Publisher, Cochrane Register of Clinical Trials, and Google scholar up to 24 August 2015. Studies were eligible if they met the following inclusion criteria: (1) design was a case–control study, retrospective or prospective follow-up study, or a randomized controlled trial (RCT); (2) the study examined the association between a genetic variant and ACL rupture in both an ACL and a control group. We determined the risk of bias for all included studies.

**Results:**

We included a total of 16 studies (eight at high risk of bias and eight with an unclear risk) that examined 33 different DNA variants. Conflicting evidence was found for the *COL1A1* rs1800012 and *COL3A1* rs1800255 variants, whereas limited evidence was found for no association of the *COL5A1* rs12722 and rs13946 and *COL12A1* rs970547 variants (all encoding collagen). Evidence was insufficient to draw conclusions as to whether any other genetic variant identified in this review had any association with ACL rupture.

**Conclusions:**

More research is needed to support a clear association between ACL rupture and genetic variants. Genome-wide studies are recommended for exploring more potential genetic variants. Moreover, large prospective studies are needed to draw robust conclusions.

## Key Points

**Table:** 

Anterior cruciate ligament (ACL) rupture is a very common and severe knee injury that predominantly occurs while participating in sports. It incurs high costs and has disastrous clinical consequences. Studies in recent years have suggested that genetic predisposition is an important factor in its etiology.
This is the first systematic review with a best-evidence synthesis regarding associations between genetic variants and ACL rupture. We found some potential genetic variants that require further investigation, especially since we identified large heterogeneity in the broad genetic variants studied and outcome definitions.
More research with large samples, phenotype homogeneity, and less bias is needed for a better understanding of the etiology of ACL rupture. This would allow us to take appropriate measures to screen for and prevent this injury and its clinical consequences.

## Introduction

An anterior cruciate ligament (ACL) rupture is a very common and severe knee injury that predominantly occurs during sports participation, primarily via a non-contact mechanism [1, 2]. An ACL rupture is often accompanied by meniscal tears (approximately 50%), medial collateral ligament injuries (22%), and chondral lesions (16–46%) and results in a tenfold increased risk of knee osteoarthritis [3–6]. As a result, an ACL rupture is referred to as ‘the stroke of the knee’ or ‘an old knee in a young patient’ [7]. ACL rupture reconstruction is one of the most commonly performed orthopedic procedures, with an increasing incidence across the globe: England (13.5 per 100,000 person-years), Scandinavian countries (32–38 per 100,000 person-years), Australia (52.0 per 100,000 person-years), and USA (43.5 per 100,000 person-years) [8–13]. In absolute numbers, this means between 100,000 and 200,000 ACL ruptures are reconstructed annually in the USA alone [13, 14]. The high incidence, high costs, and disastrous clinical consequences of ACL rupture mean it is important to be aware of the cause and mechanism behind this injury. A better understanding regarding the risk factors, etiology, and mechanism is an important step in screening for and preventing ACL rupture.

ACL rupture risk is determined by intrinsic and extrinsic factors. Extrinsic factors include the intensity of the physical activity and the type of playing surface [15–17]. Intrinsic factors include differences in anatomy, sex, neuromuscular control, and hormonal constitution [18–20]. For example, the incidence of ACL rupture is 3–6 times higher in women than in men [15, 21], which could be partially explained by the smaller intercondylar notch, higher estrogen concentration, and a movement pattern with an increased hip adductor moment and knee valgus found in women [18, 20]. Previous studies have indicated a familial predisposition for ACL rupture. An individual with an ACL rupture was twice as likely to have a relative with an ACL rupture [22]. Hewett et al. [23] pointed out that twins with an ACL rupture shared the same multiple risk factors. This might be explained by an active lifestyle, since athletes tend to injure their ACL more often than non-athletes do. However, genetics or other intrinsic variations could also be of influence.

A number of studies have suggested associations between ACL rupture and various genetic variants, possibly suggesting that genetic predisposition is a factor of importance in ACL rupture. John et al. [24] recently published a systematic review on a topic similar to ours, albeit with some notable methodological differences between the two reviews. In an attempt to conduct more sensitive research, we searched more databases. We also used a different risk-of-bias assessment tool and a best-evidence approach to synthesizing the data, which allowed us to weigh results for potential risk of bias and to grade evidence. We believe these methodological differences enabled us to generate more accurate conclusions.

To date, no systematic review with a best-evidence synthesis has been performed concerning genetics and ACL rupture. The objective of this systematic review was to summarize the current evidence for an association between genetic variants and ACL rupture.

## Methods and Materials

### Protocol

The reporting in this systematic review was conducted according to the PRISMA (Preferred Reporting Items for Systematic Reviews and Meta-Analyses) statement [25].

### Eligibility Criteria

Studies were included in the systematic review if they met the following inclusion criteria: (1) design was a case–control, retrospective or prospective follow-up study, or a randomized controlled trial; (2) the study examined the association between a genetic variant and an ACL rupture in both an ACL and a control group; (3) the study was written in English, Dutch, German, French, Spanish, Turkish, or Swedish. We excluded studies for which no full text was available, animal studies, and reviews.

### Information Sources and Search

We conducted a systematic search of the following databases up to 24 August 2015: Embase, MEDLINE, Web of Science, Scopus, PubMed Publisher, Cochrane Register of Clinical Trials, and Google scholar. The following search strategy was used in Embase: (‘anterior cruciate ligament’/de OR ‘anterior cruciate ligament injury’/de OR ‘anterior cruciate ligament rupture’/de OR (‘knee injury’/de AND (‘sports and sport related phenomena’/exp OR ‘ligament injury’/exp)) OR (‘sport injury’/de AND (knee/exp OR ‘knee ligament’/exp OR ‘ligament injury’/exp)) OR (‘anterior cruciate’ OR acl OR ((ligament*) NEAR/6 (injur* OR rupture* OR trauma* OR tear*))):ab,ti) AND (genetics/exp OR ‘genetic parameters’/exp OR (genetic* OR genom* OR gene OR genes OR (famil* NEAR/3 predispos*)):ab,ti) NOT ([animals]/lim NOT [humans]/lim). This search strategy was transferred into similar search strategies in the databases described above. References in reviews and full-text articles were screened to retrieve more studies that could be eligible for this systematic review.

### Study Selection

The results of the seven different search strategies were combined and duplicates removed using EndNoteX5. Three authors screened the results of these database searches independently by title and abstract. The final selection for inclusion of the remaining full-text articles was made by the same independent authors. Discrepancies were resolved by consensus.

### Data-Collection Process

One author extracted the general information, study design, sample size, gene, corresponding variant, and product of each study.

### Risk-of-Bias Assessment

Three reviewers, independent of each other, assessed the risk of bias of the studies using the Cochrane Centre ‘case–control tool’ [26]. Any disagreements were resolved by consensus. This risk-of-bias tool included six questions, four of which addressed bias (see Table 1). Selection bias scoring was based on the source of recruiting for cases and controls. Ideally, cases were compared with population-based controls. Confounding was scored based on age and sex. Ideally, both groups were matched or adjusted for age and sex. In addition, the studies included were scored for information bias. Ideally, the methods used to extract DNA and to genotype the genetic variant were the same. The risk of bias was divided into three ranks: low, high, and unclear risk of bias. A study was labelled ‘high risk’ if at least one bias question was answered with ‘no’ and ‘low risk’ of bias when all other questions were answered with ‘yes’. A study was labelled ‘unclear risk’ of bias if all questions were answered ‘doubtful’ or a mix of ‘doubtful’ and ‘yes’.

**Table 1:** List of questions used to assess risk of bias

#	Criterion	Question
1	Case	Are the cases defined clearly and adequately?
2	Control	Are the controls defined clearly and adequately?
3	Selection bias	Is selection bias excluded sufficiently?
4	Defined exposure	Is the exposure defined clearly, and is the method used to assess this exposure appropriate?
5	Determination	Was blinding to exposure status maintained before determination of disease?
6	Confounding	Are the main confounders identified and taken into account adequately for the design and analysis?

Information bias comprises questions 4 and 5

### Summary Measures

An overview with odds ratios (ORs) was given of various genetic variants and their associations with ACL rupture. The ACL group consisted of individuals who experienced an ACL rupture. The control group consisted of controls with no history of ACL rupture. When possible, the association with ACL rupture was examined, with subgroups being stratified according to sex and non-contact versus contact mechanism, since these factors are known to influence the risk of an ACL rupture.

### Synthesis of Results

We refrained from statistically pooling the data because of the different genetic variants and the heterogeneity of the risk of bias between studies, providing a narrative summary of the results as an alternative. Therefore, we performed a ‘best-evidence’ synthesis based on the study of van Tulder et al. [27]. Evidence was defined as generally consistent if ≥75% of the studies/cohorts reported consistent findings. Strong evidence was defined as two or more studies with a low risk of bias and generally consistent findings in all studies/cohorts. Moderate evidence was defined as one study with low risk of bias and two or more studies/cohorts with a high risk of bias and generally consistent findings. Limited evidence was defined as generally consistent findings in one study with a low risk of bias or two or more studies with a high risk of bias. Insufficient evidence was defined as a finding in one study with a high risk of bias. Conflicting evidence was defined as <75% of the studies reporting consistent findings.

## Results

### Study Selection

**Fig. 1:**
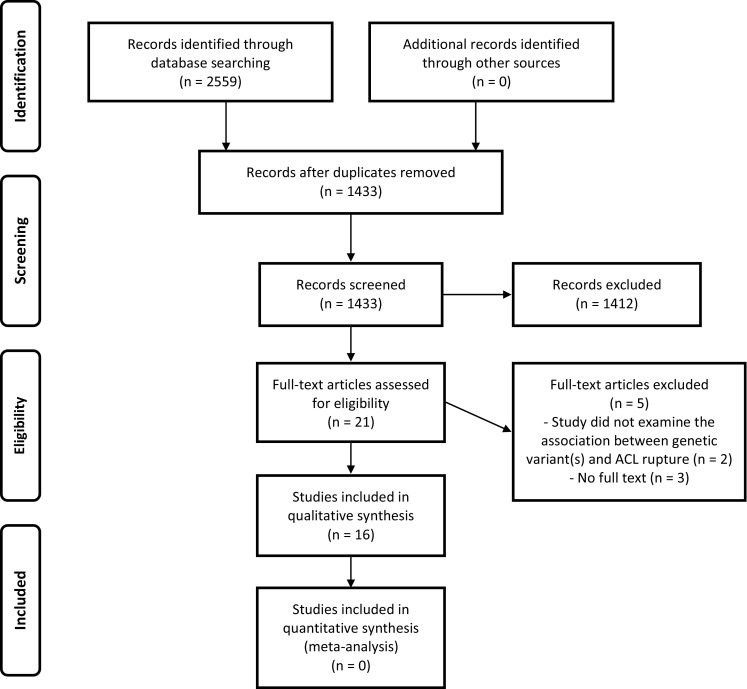
PRISMA flowchart showing the study-selection process [25]. *ACL* anterior cruciate ligament

Combining the search results of all databases retrieved a total of 2559 studies. After removing duplicates, 1433 studies remained. A further 1412 studies were excluded after screening title and abstract. Five studies were excluded after accessing the full text: two because they did not examine the association between genetic variants and ACL ruptures and three because they lacked full text. Ultimately, 16 articles fell within the scope of this systematic review. A flowchart of this process is shown in Fig. 1.

### Study Characteristics

A summary of the 16 included studies is shown in Table 2; all were case–control studies. Ten examined genetic variants in or near genes encoding collagens (collagen type I, alpha 1 [*COL1A1*]; collagen type III, alpha 1 [*COL3A1*]; collagen type V, alpha 1 [*COL5A1*]; collagen type VI, alpha 1 [*COL6A1*]; collagen type XII, alpha 1 [*COL12A1*]), one study examined proteoglycans (aggrecan [*ACAN*], biglycan [*BCN*], decorin [*DCN*], fibromodulin [*FMOD*], lumican [*LUM*]), two examined matrix metalloproteinases (matrix metalloproteinase 1 [*MMP1*], matrix metalloproteinase 3 [*MMP3*], matrix metalloproteinase 10 [*MMP10*], matrix metalloproteinase 12 [*MMP12*]), one study examined a variant near growth-differentiation factor (growth differentiation factor 5 [*GDF5*]), one investigated variants in genes involved in the angiogenesis-associated signaling cascade (vascular endothelial growth factor A [*VEGFA*], kinase insert domain receptor [*KDR*], nerve growth factor beta [*NGFB*], hypoxia-inducible factor 1-alpha [*HIF1A*]), and, finally, one study focused on elastin (*ELN*) and fibrillin (fibrillin 2 [*FBN2*]). A total of 33 different genetic variants were examined. O’Connell et al. [34] examined two different case–control cohorts in one study: South African and Polish. O’Connell et al. [34] and Ficek et al. [29] examined the *COL12A1* gene in the same population; however, O’Connell et al. [34] only performed stratified analyses, and Ficek et al. [29] analysed only the overall results. Therefore, these two studies were considered independent of each other.

**Table 2:** Study characteristics of the included studies

Study	Design	Patients with ACL rupture (n)	Controls (*n*)	Gene	Product	Variant
Ficek et al. [28] 2013	Case–control	91	143	*COL1A1*	Collagen	rs1800012
						rs1107946
Ficek et al. [29] 2014	Case–control	91	143	*COL12A1*	Collagen	rs970547
Khoschnau et al. [30] 2008	Case–control	233	325	*COL1A1*	Collagen	rs1800012
Khoury et al. [31] 2015	Case–control	141	219	*ELN*	Elastin	rs2071307
				*FBN2*	Fibrillin	rs331079
Malila et al. [32] 2011	Case–control	86	100	*MMP3*	Matrix metalloproteinase	–1612
Mannion et al. [33] 2014	Case–control	227	234	*ACAN*	Proteoglycans	rs2351491
						rs1042631
						rs1516797
				*BGN*		rs1126499
						rs1042103
				*DCN*		rs13312816
						rs516115
				*FMOD*		rs7543148
						rs10800912
				*LUM*		rs2268578
O’Connell et al. [34]^a^ 2015	Case–control	242^b^	235^b^	*COL3A1*	Collagen	rs1800255
		91^c^	91^c^	*COL6A1*		rs35796750
Posthumus et al. [35] 2009	Case–control	117	130	*COL1A1*	Collagen	rs1800012
Posthumus et al. [36] 2009	Case–control	129	216	*COL5A1*	Collagen	rs13946
						rs12722
Posthumus et al. [37] 2010	Case–control	129	216	*COL12A1*	Collagen	rs240736
						rs970547
Posthumus et al. [38] 2012	Case–control	129	216	*MMP1*	Matrix metalloproteinase	rs1799750
				*MMP3*		rs679620
				*MMP10*		rs486055
				*MMP12*		rs2276109
Rahim et al. [39] 2014	Case–control	227	227	*VEGFA*	Angiogenesis-associated signaling cascade genes	rs699947
						rs1570360
						rs2010963
				*KDR*		rs1870377
						rs2071559
				*NGFB*		rs6678788
				*HIF1A*		rs11549465
Raleigh et al. [40] 2013	Case–control	126	216	*GDF5*	Growth differentiation factor	rs143383
Stępień-Słodkowska et al. [41] 2013	Case–control	138	183	*COL1A1*	Collagen	rs1800012
Stępień-Słodkowska et al. [42] 2015	Case–control	138	183	*COL3A1*	Collagen	rs1800255
Stępień-Słodkowska et al. [43] 2015	Case–control	138	183	*COL5A1*	Collagen	rs13946
						rs12722

*ACAN* aggrecan, *BCN* biglycan, *COL12A1* collagen type XII, alpha 1, *COL1A1* collagen type I, alpha 1, *COL3A1* collagen type III, alpha 1, *COL5A1* collagen type V, alpha 1, *COL6A1* collagen type VI, alpha 1, *DCN* decorin, *ELN* elastin, *FBN2* fibrillin 2, *FMOD* fibromodulin, *GDF5* growth differentiation factor 5, *HIF1A* hypoxia-inducible factor 1-alpha, *KDR* kinase insert domain receptor, *LUM* lumican, *MMP1* matrix metalloproteinase 1, *MMP10* matrix metalloproteinase 10, *MMP12* matrix metalloproteinase 12, *MMP3* matrix metalloproteinase 3, *NGFB* nerve growth factor beta, *VEGFA* vascular endothelial growth factor A

^a^Two different cohorts were analyzed in this study, as indicated by footnote ‘b’ or ‘c’

^b^South African population

^c^Polish population

### Risk of Bias

An overview of the risk of bias is shown in Table 3. Eight studies were considered unclear risk of bias [28, 29, 36, 38, 40–43] and eight were labelled high risk of bias [30–35, 37, 39].

**Table 3:** Risk-of-bias summary: review authors’ judgements of each risk-of-bias item for each included study^a^

Study	Case (1)	Control (2)	Selection bias (3)	Defined exposure (4)	Determination exposure (5)	Confounding (6)	Overall^b^
Ficek et al. [28] 2013	+	+	?	+	?	+	?
Ficek et al. [29] 2014	+	+	?	+	?	+	?
Khoschnau et al. [30] 2008	+	?	+	+	+	−	−
Khoury et al. [31] 2015	+	+	?	+	?	−	−
Malila et al. [32] 2011	+	+	?	+	?	−	−
Mannion et al. [33] 2014	+	+	?	+	?	−	−
O’Connell et al. [34] 2015	+	?	?	+	+	−	−
Posthumus et al. [35] 2009	+	+	?	+	?	−	−
Posthumus et al. [36] 2009	+	+	?	+	?	+	?
Posthumus et al. [37] 2010	+	+	?	+	?	−	−
Posthumus et al. [38] 2012	+	+	?	+	?	+	?
Rahim et al. [39] 2014	+	?	?	+	?	-	−
Raleigh et al. [40] 2013	+	+	?	+	?	+	?
Stępień-Słodkowska et al. [41] 2013	+	+	?	+	?	?	?
Stępień-Słodkowska et al. [42] 2015	+	+	?	+	?	?	?
Stępień-Słodkowska et al. [43] 2015	+	+	?	+	?	?	?

^a^Numbers 1–6 in the column headings correspond to the questions listed in Table 1; − indicates the risk of bias question was answered ‘no’, + indicates the risk of bias question was answered ‘yes’, ? indicates the risk of bias question could not be answered either ‘yes’ or ‘no’ and was answered with a ‘doubtful’ or ‘unknown’

^b^− indicates a high risk of bias, ? indicates an unclear risk of bias

### Results of Association Studies in the Complete Populations

Results of the association studies are shown in Table 4.

**Table 4:** Results of genetic studies examining associations between genetic variants and anterior cruciate ligament rupture

Study	Gene	Protein	Variant	Genetic analysis	OR (95% CI)	*p*-Value	Risk of bias
Ficek et al. [28] 2013	*COL1A1*	Collagen type I	rs1800012	GG vs. GT + TT	Not shown	>0.05	Unclear
				GT vs. GG + TT	Not shown	>0.05	
				TT vs. GG + GT	Not shown	>0.05	
			rs1107946	GG vs. GT + TT	Not shown	>0.05	
				GT vs. GG + TT	Not shown	>0.05	
				TT vs. GT + GG	Not shown	>0.05	
Ficek et al. [29] 2014	*COL12A1*	Collagen type XII	rs970547	GG vs. GA + AA	Not shown	>0.05	Unclear
				GA vs. GG + AA	Not shown	>0.05	
				AA vs. GG + GA	Not shown	>0.05	
Khoschnau et al. [30] 2008	*COL1A1*	Collagen type I	rs1800012	GG vs. GG	1	>0.05	High
				GT vs. GG	1.19 (0.82–1.75)	>0.05	
				TT vs. GG	0.12 (0.02–0.92)	**<0.05** ^d^	
Khoury et al. [31] 2015	*ELN*	Elastin	rs2071307	GG vs. GA + AA	Not shown	>0.05	High
				GA vs. GG + AA	Not shown	>0.05	
				AA vs. GG + GA	Not shown	>0.05	
	*FBN2*	Fibrillin-2	rs331079	GG vs. GC + CC	Not shown	>0.05	
				GC vs. GG + CC	Not shown	>0.05	
				CC vs. GG + GC	Not shown	>0.05	
Malila et al. [32] 2011	*MMP3*	Matrix metalloproteinase type 3	–1612	5A+ vs. 5A−	1.39 (0.72–2.67)	>0.05	High
				5A− vs. 5A+	0.72 (0.37–1.38)	>0.05	
Mannion et al. [33] 2014	*DCN*	Decorin	rs516115	GG vs. GA + AA	9.23 (1.17–73.01)^e^	**0.015**	High
				GA vs. GG + AA	Not shown	>0.05	
				AA vs. GG + GA	Not shown	>0.05	
			rs13312816	AA vs. AT + TT	Not shown	>0.05	
				AT vs. AA + TT	Not shown	>0.05	
				TT vs. AA + AT	Not shown	>0.05	
	*ACAN*	Aggrecan	rs2351491	CT vs. CC + TT	Not shown	>0.05	
				CC vs. CT + TT	Not shown	>0.05	
				TT vs. CT + CC	Not shown	>0.05	
			rs1042631	TT vs. CT + CC	Not shown	>0.05	
				CT vs. TT + CC	Not shown	>0.05	
				CC vs. CT + TT	Not shown	>0.05	
			rs1516797	TT vs. GT + GG	Not shown	>0.05	
				GT vs. GG + TT	Not shown	>0.05	
				GG vs. GT + TT	Not shown	>0.05	
	*BGN*	Biglycan	rs1126499	CC vs. CT + TT	Not shown	>0.05	
				CT vs. CC + TT	Not shown	>0.05	
				TT vs. CC + CT	Not shown	>0.05	
			rs1042103	GG vs. GA + AA	Not shown	>0.05	
				GA vs. GG + AA	Not shown	>0.05	
				AA vs. GG + GA	Not shown	>0.05	
	*FMOD*	Fibromodulin	rs7543148	GG vs. GA + AA	Not shown	>0.05	
				GA vs. GG + AA	Not shown	>0.05	
				AA vs. GG + GA	Not shown	>0.05	
			rs10800912	CC vs. CT + TT	Not shown	>0.05	
				CT vs. CC + CT	Not shown	>0.05	
				TT vs. CC + CT	Not shown	>0.05	
	*LUM*	Lumican	rs2268578	TT vs. TC + CC	Not shown	>0.05	
				TC vs. TT + CC	Not shown	>0.05	
				CC vs. CT + TT	Not shown	>0.05	
O’Connell et al. [34]^a^ 2015	*COL3A1*	Collagen type III^b^	rs1800255	AA vs. GA + GG	Not shown	>0.05	High
				GA vs. GG + AA	Not shown	>0.05	
				GG vs. AA + GA	Not shown	>0.05	
		Collagen type III^c^		AA vs. GA + GG	3.8 (1.1–12.8)	**0.036**	
				GA vs. AA + GG	Not shown	>0.05	
				GG vs. AA + GA	Not shown	>0.05	
		Collagen type VI^b^	rs35796750	TT vs. TC + CC	Not shown	>0.05	
				TC vs. CC + TC	Not shown	>0.05	
				CC vs. TT + CT	Not shown	>0.05	
Posthumus et al. [35] 2009	*COL1A1*	Collagen type I	rs1800012	TT vs. GT + GG	0.08 (<0.01–1.46)	**0.031**	High
				GT vs. TT + GG	Not shown	>0.05	
				GG vs. GG + GT	Not shown	>0.05	
Posthumus et al. [36] 2009	*COL5A1*	Collagen type V	rs12722	TT vs. CT + CC	Not shown	>0.05	Unclear
				CT vs. TT + CC	Not shown	>0.05	
				CC vs. TT + CT	Not shown	>0.05	
			rs13946	TT vs. CT + CC	Not shown	>0.05	
				CT vs. TT + CC	Not shown	>0.05	
				CC vs. TT + CT	Not shown	>0.05	
Posthumus et al. [37] 2010	*COL12A1*	Collagen type XII	rs970547	GG vs. GA + AA	Not shown	>0.05	High
				GA Vs. AA + GG	Not shown	>0.05	
				AA VS. GG + GA	Not shown	>0.05	
			rs240736	TT vs. CT + CC	Not shown	>0.05	
				CT vs. TT + CC	Not shown	>0.05	
				CC vs. TT + CT	Not shown	>0.05	
Posthumus et al. [38] 2012	*MMP1*	Matrix metalloproteinase type 1	rs1799750	CC vs. CT + TT	Not shown	>0.05	Unclear
				CT vs. CC + TT	Not shown	>0.05	
				TT vs. CC + CT	Not shown	>0.05	
	*MMP3*	Matrix metalloproteinase type 3	rs679620	1G1G vs. 1G2G + 2G2G	Not shown	>0.05	
				1G2G vs. 1G1G + 2G2G	Not shown	>0.05	
				2G2G vs. 1G1G + 1G2G	Not shown	>0.05	
	*MMP10*	Matrix metalloproteinase type 10	rs486055	GG vs. GA + AA	Not shown	>0.05	
				GA vs. GG + AA	Not shown	>0.05	
				AA vs. GG + GA	Not shown	>0.05	
	*MMP12*	Matrix metalloproteinase type 12	rs2276109	AA vs. AG + GG	Not shown	>0.05	
				AG vs. AA + GG	Not shown	>0.05	
				GG vs. AA + AG	Not shown	>0.05	
Rahim et al. [39] 2014	*VEGFA*	Vascular endothelial growth factor A	rs699947	CC vs. CA + AA	Not shown	>0.05	High
				CA vs. CC + AA	Not shown	>0.05	
				AA vs. CC + CA	Not shown	>0.05	
			rs1570360	GG vs. GA + AA	Not shown	>0.05	
				GA vs. GG + AA	1.70 (1.16–2.50)	**0.007**	
				AA vs. GG + GA	Not shown	>0.05	
			rs2010963	GG vs. GC + CC	Not shown	>0.05	
				GC vs. GG + CC	Not shown	>0.05	
				CC vs. GC + GG	Not shown	>0.05	
	*KDR*	Kinase insert domain receptor	rs1870377	TT vs. TA + AA	Not shown	>0.05	
				TA vs. TT + AA	Not shown	>0.05	
				AA vs. TT + AT	Not shown	>0.05	
			rs2071559	GG vs. GA + AA	Not shown	>0.05	
				GA vs. GG + AA	Not shown	>0.05	
				AA vs. GA + GG	Not shown	>0.05	
	*NGFB*	Nerve growth factor beta	rs6678788	CC vs. CT + TT	Not shown	>0.05	
				CT vs. GG + TT	Not shown	>0.05	
				TT vs. GT + CC	Not shown	>0.05	
	*HIF1A*	Hypoxia-inducible factor 1-alpha	rs11549465	CC vs. CT + CC	Not shown	>0.05	
				CT vs. CC + TT	Not shown	>0.05	
				TT vs. CT + CC	Not shown	>0.05	
Raleigh et al. [40] 2013	*GDF5*	Growth-differentiation hormone factor	rs143383	TT vs. CT + CC	Not shown	>0.05	Unclear
				CT vs. TT + CC	Not shown	>0.05	
				CC vs. TT + CT	Not shown	>0.05	
Stępień-Słodkowska et al. [41] 2013	*COL1A1*	Collagen type I	rs1800012	GG vs. GT + TT	Not shown	**0.046**	Unclear
				GT vs. GG + TT	Not shown	>0.05	
				TT vs. GT + GG	Not shown	>0.05	
Stępień-Słodkowska et al. [42] 2015	*COL3A1*	Collagen type III	rs1800255	GG vs. GA + AA	0.78 (0.49–1.24)	>0.05	Unclear
				GA vs. GG + AA	Not shown	>0.05	
				AA vs. GG + GA	5.05 (1.62–15.78)	**0.003**	
Stępień-Słodkowska et al. [43] 2015	*COL5A1*	Collagen type V	rs13946	CC vs. CT + TT	Not shown	>0.05	Unclear
				CT vs. CC + TT	Not shown	>0.05	
				TT vs. CC + CT	Not shown	>0.05	
			rs12722	CC vs. CT + TT	Not shown	>0.05	
				CT vs. CC + TT	Not shown	>0.05	
				TT vs. CC + CT	Not shown	>0.05	

Bold type indicates statistical significance (*p* < 0.05)

*ACAN* aggrecan, *ACL* anterior cruciate ligament, *BCN* biglycan, *COL12A1* collagen type XII, alpha 1, *COL1A1* collagen type I, alpha 1, *COL3A1* collagen type III, alpha 1, *COL5A1* collagen type V, alpha 1, *COL6A1* collagen type VI, alpha 1, *DCN* decorin, *ELN* elastin, *FBN2* fibrillin 2, *FMOD* fibromodulin, *GDF5* growth differentiation factor 5, *HIF1A* hypoxia-inducible factor 1-alpha, *KDR* kinase insert domain receptor, *LUM* lumican, *MMP1* matrix metalloproteinase 1, *MMP10* matrix metalloproteinase 10, *MMP12* matrix metalloproteinase 12, *MMP3* matrix metalloproteinase 3, *NGFB* nerve growth factor beta, *OR* odds ratio, *VEGFA* vascular endothelial growth factor A

^a^Two different cohorts were analysed in this study, as indicated by footnote ‘b’ or ‘c’

^b^South African group

^c^Polish group

^d^No exact *p*-value was reported

^e^This genotype was over-represented (OR = 9.23) in the control group compared with the ACL group, which is consistent with a protective effect in the ACL group (OR = 1/9.23 = 0.11)

#### Collagen

The most frequently studied gene was *COL1A1*. Conflicting evidence was found for an association between TT and GG genotype of the *COL1A1* rs1800012 variant and ACL rupture. Conflicting evidence was found for an association between AA genotype of the *COL3A1* rs1800255 variant and ACL rupture. Limited evidence was found for no association between *COL5A1* rs12722, *COL5A1* rs13946, and *COL12A1* rs970547 variants and ACL rupture. Insufficient evidence was found for no association between *COL1A1* rs1107946, *COL6A1* rs35796750, and *COL12A1* rs240736 variants and ACL rupture.

#### Proteoglycans

Insufficient evidence was found for an association between GG (protective) genotype of the *DCN* rs516115 variant and ACL rupture. Insufficient evidence was found for no association between *DCN* rs13312816, *ACAN* rs2351491, *ACAN* rs1042631, *ACAN* rs1516797, *BGN* rs1126499, *BGN* rs1042103, *FMOD* rs7543148, *FMOD* rs10800912, and *LUM* rs2268578 variants and ACL rupture.

#### Matrix Metalloproteinases

Insufficient evidence was found for no association between *MMP1* rs1799750, *MMP3* rs679620, *MMP3*-1612, *MMP10* rs486055, and *MMP12* rs2276109 variants and ACL rupture.

#### Angiogenesis-Associated Signaling Cascade and Growth Differentiation Hormone Factor

Insufficient evidence was found for an association between GA genotype (harmful) of the *VEGFA* rs1570360 variant and ACL rupture. Insufficient evidence was found for no association between *VEFGA* rs699947, *VEFGA* rs2010963, *KDR* 1870377, *KDR* rs2071559, *NGFB* rs6678788, *HIF1A* rs11549465, and *GDF5* rs143383 variants and ACL rupture.

#### Elastin and Fibrillin

Insufficient evidence was found for no association between *ELN* rs2071307 variant and ACL rupture or for no association between *FBN2* rs331079 variant and ACL rupture.

### Stratified Analysis

In addition to the overall analyses, studies investigated genetic variants in sex and/or (non-) contact stratified analyses. However, because of the small sample sizes, insufficient data were available to report sufficient evidence regarding those analyses. Therefore, they were not included in this review.

## Discussion

In this systematic review, we summarized the current literature on genetic variants predicting the risk of ACL rupture. We found conflicting evidence for the *COL1A1* rs1800012 GG and TT genotype and *COL3A1* rs1800255 AA genotype and limited evidence for no association between *COL5A1* rs13946, *COL5A1* rs12722, and *COL12A1* rs970547 variants and ACL rupture. We also found associations, albeit with insufficient evidence, regarding the *DCN* rs516115 GG genotype (protective) and *VEGFA* rs1570360 GA genotype (harmful) and ACL rupture. Moreover, a large number of genetic variants were found not to have an association. However, those genetic variants were studied only once; therefore, evidence for those DNA variants was also considered insufficient. We included 16 studies in this review, with a total of 33 different genetic variants. Many studies were found to have an unclear or high risk of bias and confounding. In addition, we identified large heterogeneity in the genetic variants studied, outcome definition, and the genetic contrast studied, which made it impossible to conduct a formal meta-analysis of these studies. Therefore, we performed a best-evidence synthesis. Overall, we found some potential genetic variants that could influence the risk of ACL rupture. However, more data are needed to support a clear association between genetic variants and ACL rupture. Larger and more genetic studies are required to obtain a better understanding of these possible associations.

John et al. [24] recently published a similar systematic review. However, there are some notable differences between the two studies. First, John et al. [24] presented the results as a narrative review and concluded that, of the 20 genes examined, ten were positively associated with an ACL rupture. Their review does not appear to fully justify their finding that 50% (*COL1A1*, *COL12A1*, *COL5A1*, *COL3A1*, *MMP3*, *MMP12*, and various *ECM*) of the genes examined so far are positively associated with an ACL tear, especially when mentioning contradictory results for some specific genetic variants such as *COL1A1* or *COL3A1*. The current analysis presented the findings using a best-evidence synthesis by van Tulder et al. [27]. A best-evidence synthesis provides stronger evidence and takes a different approach to presenting the results than does a simple narrative summary. Consequently, the results and conclusions of the current analysis concerning the associations between genetic variants and ACL injury differ from and have greater methodological power than those of John et al. [24]. Second, this review included two additional studies [30, 40]. John et al. [24] excluded one of these [30] because both the ACL and the posterior cruciate ligaments were included and analysed together in one population group. They also excluded a different study [40], likely because of differences between our search strategies and because this review searched more databases. Third, in contrast to John et al. [24], we did not account for subgroups such as sex and injury mechanism because of small sample sizes. Fourth, this review concentrated on polymorphisms rather than less well-studied haplotypes or alleles. Fifth, John et al. [24] did not report the results of genetic variants for which no association was found with ACL rupture. This review represents a survey of all investigated genetic variants and genotypes with their ORs and p-values, even if no association was found at all. This approach was taken in the interests of maximum transparency and clarity, allowing readers and researchers to decide which genetic variants to investigate in the future, taking into account confidence intervals and statistical significance data.

Variants of the investigated genes, displayed in Table 4, are involved in the synthesis, strength, and homeostasis of the ligament. *COL1A1* encodes for collagen type I, which provides mechanical strength to several tissues, including ligaments [44]. *COL3A1* encodes for collagen type III and is involved in collagen type I fibrillogenesis [45]. *COL5A1* encodes collagen type V, which is engaged with collagen type I in constructing heterotypic fibrils and also regulates the diameter of those fibrils [46]. Collagen type XII, encoded by *COL12A1*, is the largest member of the fibril-associated collagens and regulates the organization and mechanical properties of collagen fibril bundles [47]. Decorin, encoded by the *DCN* gene, belongs to the small group of proteoglycans and is engaged in limiting the diameter of collagen fibrils during fibrillogenesis [48]. *VEGFA* encodes vascular endothelial growth factor A, is a regulator of angiogenesis, and increases the expression of the matrix metalloproteinases [49]. The consequences of those genetic variants are not exactly known. To date, a limited number of the genetic variants involved in the synthesis, strength, and homeostasis of the ligament have been investigated. Moreover, most of those genetic variants were only studied once. Only some genetic variants in or near *COL1A1* (4x), *COL3A1* (3x), *COL5A1* (2x), and *COL12A1* (2x) were studied in more than one independent study/cohort.

Our included studies had some limitations. No limit was set on minimum sample size to enable us to include all possible studies because genetic studies require more participants than most of our included studies had. Sample size became very small when groups were stratified according to sex or mechanism of injury and, therefore, we did not report any stratified analyses. In total, 14 published studies used, partially or fully, the same population for cases and controls [28, 29, 31, 33–43], which increased the risk of bias. Most likely, every gene variant has its own potential, influencing the risk of an ACL rupture. For example, the *VEGFA* rs1570360 and *DCN* rs516115 variants were examined in nearly the same population group, which was overrepresented in the ACL rupture group [33, 39]. While both of these genetic variants could have increased the risk of ACL rupture, the possibility remains that only one of them was the actual risk contributor while the association of the other genetic variant was modified (confounding or effect modification) by the actual risk variant. Thus, another limitation is possible: confounding by ethnicity (population stratification). If the risk of an ACL rupture differs between different ethnicities but there is also a variation in the statistical distribution of a genetic variant between ethnic groups, the association between the ACL rupture and genetic variant may be confounded by the ethnic background of the studied population [50].

The heterogeneity of several study aspects, such as differences in population and type of genetic variants, meant that a meta-analysis was not possible. Therefore, we performed a best-evidence synthesis as an alternative. All of our studies were case-controlled as it is nearly impossible, and unnecessary, to conduct a randomized controlled trial given the research question. However, prospective cohort studies would provide stronger evidence.

Another major issue remains the possible underlying heterogenetic etiology in genetic studies, for example in patients with osteoarthritis [51]. Therefore, future research should also focus on evaluating larger samples and resolving phenotype heterogeneity to facilitate more comprehensive study of the genetics of ACL rupture, for example by investigating genetic variants in established genome-wide studies conducted to assess other factors such as osteoarthritis [51–54].

In the risk-of-bias assessment, eight studies were considered at high risk of bias. Eight studies were labelled at unclear risk of bias, which exemplifies the quality of the research and the conclusions that can be distilled.

Research has already been conducted for various genes involved in production, strength, or homeostasis of the ACL. However, as mentioned, larger and more genetic studies are required to provide a better understanding of the possible associations with ACL rupture. Every genetic variant examined should be re-examined to obtain a better understanding of the influence of these genes. Furthermore, research is encouraged for collagen and matrix metalloproteinase genes other than those already studied [55, 56]. Tendons and ligaments largely share the same components and both belong to the soft tissues. A systematic review by Claessen et al. [57] indicated no association between the *COL1A1* rs1800012 variant and Achilles tendon ruptures, which does not clarify whether ACL and Achilles tendon ruptures might share the same genetic risk factors. More variants found in tendon ruptures, such as the *TIMP* gene, should also be investigated in relation to ACL ruptures [57]. This also applies to genetic variants found in osteoarthritis, since ACL rupture is a major risk factor for osteoarthritis [52, 53].

Some studies addressed the interaction between two gene variants on one chromosome, called haplotypes, which were found to modify the risk of an ACL rupture [28, 33, 34, 38, 39]. If genetic variants were not found to influence the risk, an association was still found due to the haplotype of those two gene variants. More research would be needed to clarify the exact role of haplotypes.

The examination of ACL rupture genetics is valuable, since knowledge of the genetic variants involved could contribute to an understanding of the etiology and risk factors in ACL tears. In addition, genetic variants could help in screening and prevention. Each person has a unique genetic profile. Some studies already suggest using genetic profiles to enhance athletic performance [58, 59]. Taking appropriate preventive measures might decrease the risk of an ACL rupture, as well as its costs [60, 61].

Screening for genetic variants could be implemented in different scenarios. Professional sports organizations might take appropriate measures regarding players at high risk of an ACL rupture. High-risk players could decide at a young age not to start a career in high-risk sports. Furthermore, screening could be implemented in families with an active lifestyle if a first-degree relative has a history of an ACL rupture. However, in reality, screening for risk of ACL rupture would be a complex task. It should be noted that none of the genetic variants solely influence the risk and thus could not be used as a diagnostic tool in isolation. As previously stated, an ACL rupture is determined by various intrinsic and extrinsic factors in which each factor contributes a small amount to the risk. Therefore, multifactorial and comprehensive models are designed to predict the risk of ACL rupture [62]. In future, when an association with genetics is found, genetic risk factors might be included in the current multifactorial models predicting the risk of ACL rupture [63]. Appropriate screening and prevention programs might be implemented based on those models. Individuals understand, and are interested in, the benefits of genomic testing in psychological and medical terms [64]. Unfortunately, genetic testing remains a source of moral and ethical controversy [64, 65].

## Conclusion

More evidence is needed to draw significant conclusions regarding the association between genetic variants and ACL rupture. We did find some genetic variants that potentially contribute. Conflicting evidence was found for *COL1A1* rs1800012 and *COL3A1* rs1800255, whereas limited evidence was found for no association with *COL5A1* rs13946, *COL5A1* rs12722, and *COL12A1* rs970547. Finally, we found insufficient evidence for an association between ACL rupture and *DCN* rs516115 GG genotype (protective) or *VEGFA* rs1570360 GA genotype (harmful). The genetic variants included in this systematic review account for only a small number of the genes involved in the biology of the ligament. ACL rupture has a high incidence and incurs extreme consequences. Therefore, more high-quality and homogenous data are needed to provide a better understanding of the etiology, which in future might improve screening and prevention programs. Research should primarily focus on the components and homeostasis of the ligament. These future genetic investigations should be performed in large (collaborative) genome-wide association studies with large sample sizes and phenotype homogeneity to explore for more potential genetic variants. However, and more importantly, future research should focus on (large) prospective studies so that clinically significant conclusions can be drawn.
